# 

**Published:** 1890-08

**Authors:** 


### LITERARY.

THE SUPPRESSION OF CONSUMPTION. By G. W. Hambleton, M. D., President Polytechnic Physical Development of Great Britain.

This is a pamphlet of 37 pages, published by N. D. C. Hodges, 47 Lafayette Place, New York city.

The author in his preface, in speaking of America, says, "Her great cities are the centres of consumption, and far away from them, on the borders of advancing civilization, 'Nature's Cures' have been frequently accomplished. A great advance in science has been made. We now know in what both processes consist, and the responsibility for the suppression of consumption rests with us."

This is a frank avowal of the relation in which the medical profession stand to the worst of our pulmonary diseases. The text is clear as to the causes and means of cure of this usually fatal malady, with the particulars of condition and treatment of no less than twenty-two individual cases.

The treatise is a candid exposition of the author's theory and practice with consumptives in the various stages of the disease, and well worthy the attention of the medical faculty.

THE ESOTERIC.—We have on more than one occasion called attention to the excellence of this magazine. The editorial management is now committed to C. H. Mackay, Esq., well known as a writer of great research and ability. Published monthly at 478 Shawmut Avenue, Boston.

RECOLLECTIONS OF GENERAL GRANT; by Geo. W. Childs. Any thing reliable in relation to the foremost soldier of the North, who directed the course of armies and re-established the Union of States, belongs to history, and Mr. Childs has laid his country under new obligations, by giving us these faithful chapters of his ways and methods in the midst of civil conflict, and the retirement of private life. The chapters relating to West Point, and the presentation there by the author, of the portraits of the three great Captains who forced the destiny of the Republic with a single impulse, find a fitting place in these pages.

Of Mr. Childs, it is not too much to say that his name and reputation for generous gifts, freely and kindly bestowed, extend the world over. Indeed, no American in private life is better known or more highly esteemed. Long life to him and days full of blessings.

REVUE INTERNATIONALE DE BIBLIOGRAPHIE, Médicale, Pharmaceutique et Vétérinaire, directed by Dr. Jules Rouvier, Professor of Medical Science, etc., etc. Paris, 23 Rue Racine, and Beyrouth, Syria.

This is No. 2 of the first volume of a most thorough catalogue of medical publications, in nearly all the countries of the globe, where medicine and surgery are recognized and scientifically practiced in the healing of wounds and the cure of diseases. The two volumes contain about 600 closely printed pages of valuable information upon the subject in hand.

PRACTICAL SANITARY AND ECONOMIC COOKING.—By Mrs. Mary Hinman Abel; the first prize essay of the American Public Health Association, through the liberality of Mr. Henry Lomb, of Rochester, N. Y.; published by the Association. Irving A. Watson, Secretary, Concord, N. H.

This handy little volume of 190 pages of facts and methods, and recipes for cooking nearly all the requisite family dishes, is a gratifying return for the liberal prize of $500, offered by Mr. Lomb, and may well serve as an economic guide in the culinary branch of many a household, where healthy and inexpensive living are objects to be attained, for "Good digestion waits on appetite, and health on both."

A STORY OF DAMON AND PYTHIAS.—Souvenir to the Knights of Pythias of the world; so says the title page, and it ought to know.

The story belongs to classic lore and is familiar to every schoolboy. There are six photo-engravings, five, of the relics of ancient grandeur in the high old days of barbaric splendor, when Dionysius held undisputed sway in Sicily's ancient capital. The sixth, is a bird's-eye view of Pobst's brewing establishment in Milwaukee; why should they not go together? Dionysius was a liberal patron of the rich juices of the Sicilian grape, and had Pobst's brewery been in operation there who knows what might have happened?

Let the rotund corporations of Teutonic indulgence leave it to be imagined. As requested, we send a copy of this to the aforesaid Company and await our complimentary keg. Here's to Pobst, meanwhile, in weak ice-water.

We are under obligations to the various authors for the following treatises, etc.

INTESTINAL ANASTOMOTIC OPERATIONS WITH SEGMENTED RUBBER RINGS; by A. V. L. Brokaw, M. D., Demonstrator of Anatomy, etc., St. John's Hospital, St. Louis, Mo.

TWENTY CASES OF ABDOMINAL SECTION; by L. S. McMurtry, M. D., Louisville, Ky.

ELECTROLYSIS IN THE TREATMENT OF OBSTRUCTION, ETC.; by Robert Newman, M. D., Hospital Surgeon, etc.

APPARENT CANCEROUS TRANSFORMATION OF SYPHILOMA OF THE TONGUE; by G. Frank Lydston, M. D., Chicago, Ill.

Also the following:

Announcement of the 18th Annual Meeting of the American Health Association, to be held at Charleston, S. C., in December, 1890.

Abstract of the 1st Annual Report of the State Commission in Lunacy.

Report of Intercollegiate Committee of the American Institute of Homœopathy, 1890.

**TUSKEGEE, ALABAMA, STATE NORMAL AND INDUSTRIAL INSTITUTE.—This highly creditable institution was opened July 4, 1881, in a church building, with one teacher and 30 students. Now, in its eighth year, it has 27 officers and teachers and 425 students. There is a military training school connected with the institute that carries the old flag. Of course, it signifies one country, one flag and one destiny, now and forever.**

